# Treated Primary Cutaneous Malignant Melanoma With Later Metastasis Found in Clinical Presentation of Left Axilla Lymphadenopathy: A Case Report

**DOI:** 10.7759/cureus.54694

**Published:** 2024-02-22

**Authors:** Brigitte L Cochran, Sara Eliseo, Austin Vaughn, Tamryn L Van Der Horn, Enzo Ferrara, Jamie Edwards

**Affiliations:** 1 Osteopathic Medicine, Philadelphia College of Osteopathic Medicine, Moultrie, USA; 2 Radiology, Lake Erie College of Osteopathic Medicine, Bradenton, USA; 3 Osteopathic Medicine, Philadelphia College of Osteopathic Medicine, Suwanee, USA; 4 Interventional Radiology, St. Vincent's Medical Center, Jacksonville, USA

**Keywords:** axillary lymphadenopathy, ultrasound-guided, h&e staining, sox 10, cutaneous malignant melanoma, malignant melanoma metastasis, intraductal carcinoma

## Abstract

This case report details the rare instance of metastatic spread of cutaneous malignant melanoma to the breast in a 50-year-old female. The patient presented with a palpable axillary mass confirmed to be metastasis despite excision and closure of the primary malignancy. The mass seen in clinical and radiological presentations presented with features of complicated differentiation from a primary breast tumor. Biopsy and staining with immunohistochemical markers S100 and Sox10 played a critical role in confirming the melanocytic origin of this metastatic lesion. Breast metastases are associated with poor prognosis, and this case emphasizes the importance of in-depth evaluations for patients with a history of malignant melanoma and the need for ongoing clinical awareness in this field.

## Introduction

Malignant melanoma is one of the most deadly malignancies worldwide, with most arising from primary skin, ocular, or mucosal sites [1]. Melanoma only represents 1-2% of recorded malignancies, but once encountered, it can be deadly [2]. Malignant melanoma has two manifestations: cutaneous and non-cutaneous. Cutaneous melanomas make up the majority of melanomas encountered in practice with the most common resulting from exposure to UV radiation [2]. There are many other factors in play, besides UV radiation exposure that influence the metastatic potential of cutaneous melanomas. Some include inherited genetic mutations affecting BRAF or NRAS, family history of melanoma, disturbances to the immune system, and the skin tone of the individual [2]. Unlike cutaneous melanoma, noncutaneous melanoma is not related to the amount of sun exposure an individual may have [3]. Noncutaneous melanomas also show poorer prognostic outcomes than cutaneous melanomas due to their late clinical presentation and poor response to immunotherapy [3,4]. This complex group of malignancies is most commonly seen in the form of ocular and mucosal melanomas, but noncutaneous melanomas can also be found in the adrenal glands, meninges, and breasts [5].

Primary melanoma of the breast accounts for less than 5% of all melanomas [2]. Noncutaneous melanoma of the breast is exceedingly rare, being documented as only consisting of 0.5% of all breast cancers [2]. This subtype of noncutaneous melanoma has a high mortality and poor prognostic outcome due to the increased likelihood of metastasis to distant organs and lymph nodes [2].

This case report discusses a patient who presented with primarily cutaneous malignant melanoma located on the left breast that was previously treated and later metastasized to the left axillary lymph node through confirmed ultrasound imaging and biopsy.

## Case presentation

A 50-year-old female presented to her primary care physician with a palpable lump found on self-examination of the left axilla that was present for one to two weeks. The patient denied new cutaneous lesions as well as chest, bone and abdominal pain. The patient also denied recent unintentional weight loss and palpable breast masses. This patient had a history of cutaneous stage 1 malignant melanoma, per the tumor, node, and metastasis (TNM) staging system by the American Joint Committee on Cancer (AJCC), located on the left breast and was four years overdue for her annual mammogram when encountered in the clinic. During a physical examination, a 6 mm cystic mass was palpated under the left axilla, therefore a diagnostic ultrasound and biopsy was done to rule out malignancy and/or metastasis of the left axilla lesion.

**Figure 1:**
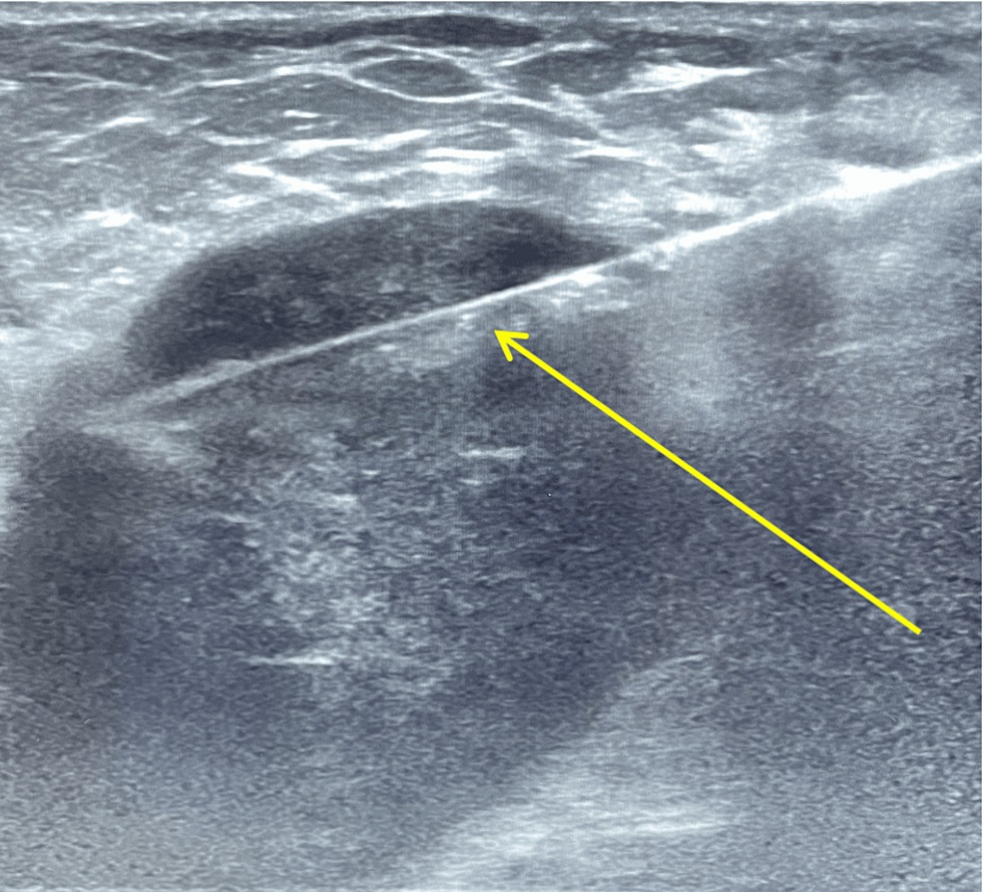
Ultrasound imaging of the mass located within the left axilla, undergoing image-guided core needle biopsy, with the needle piercing the mass pictured (yellow arrow).

The skin over the entry site of the left axilla was cleansed and draped in a protocol sterile environment. The patient was given local anesthesia with 1% lidocaine. The lesion was biopsied via ultrasound guidance, in which a coaxial needle was advanced to the edge of the mass and a 16-gauge core needle biopsies were obtained (Figure 1). A biopsy clip was placed in the central portion of the mass. Core samples of 6 x 16-gauge from the left mass were submitted for anatomic pathology and flow cytometry. Specimens were received in formalin solution with the patient’s name and the location of the “left axilla”. 

**Figure 2:**
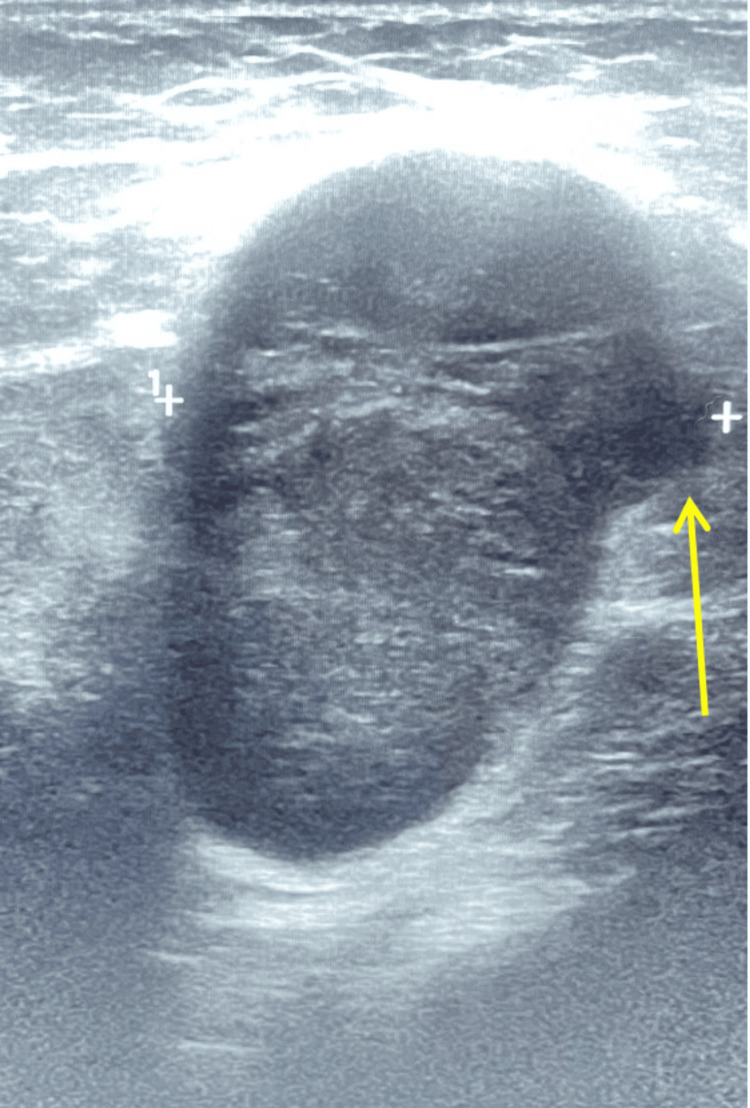
Left axilla hypoechoic mass with well-circumscribed wall but with an irregular border protrusion located on the right upper aspect pictured with + sign (yellow arrow).

**Figure 3:**
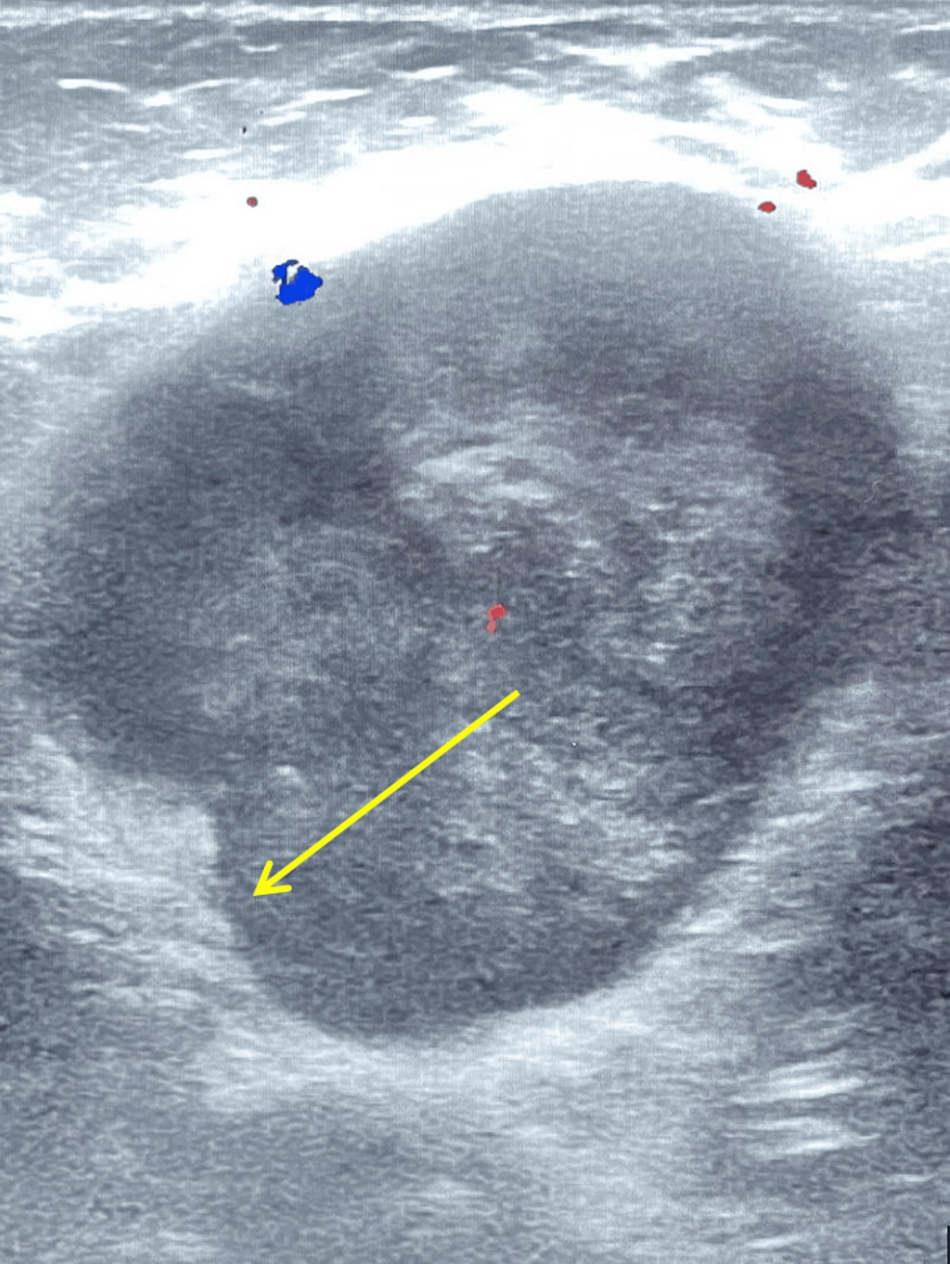
A sagittal view of the left axilla mass, in which further irregularity of the borders is confirmed in the left lower quadrant (yellow arrow).

Ultrasound analysis demonstrated a hypoechoic left axillary mass measuring 4.7 x 3.9 x 3.4 cm that was read by radiology on September 8, 2023 (Figures 2, 3). 

**Figure 4:**
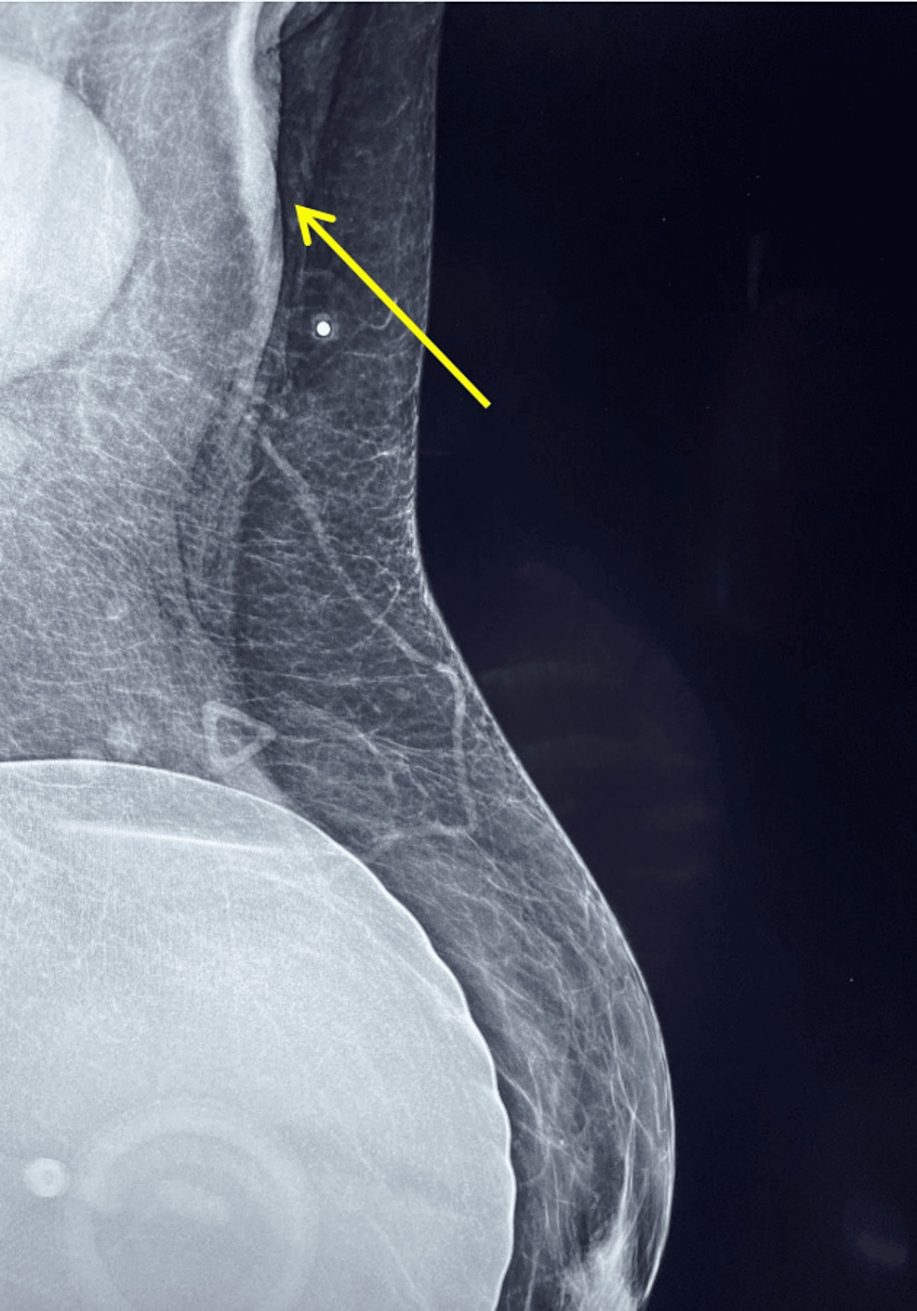
A mammogram that was obtained on August 28, 2023, with a medial to lateral view of the right breast, with the enlarged left lymph node located on the right superior aspect of the figure. Silicone implants are noted in the left lower area and the palpable lymph node is pictured in the upper left quadrant (yellow arrow).

Specimens submitted were three white-yellow fibrofatty tissue cores, measuring 1.2-1.5 cm. A mammogram was documented that illustrated the clinical presentation of axillary mass (Figure 4).

**Figure 5:**
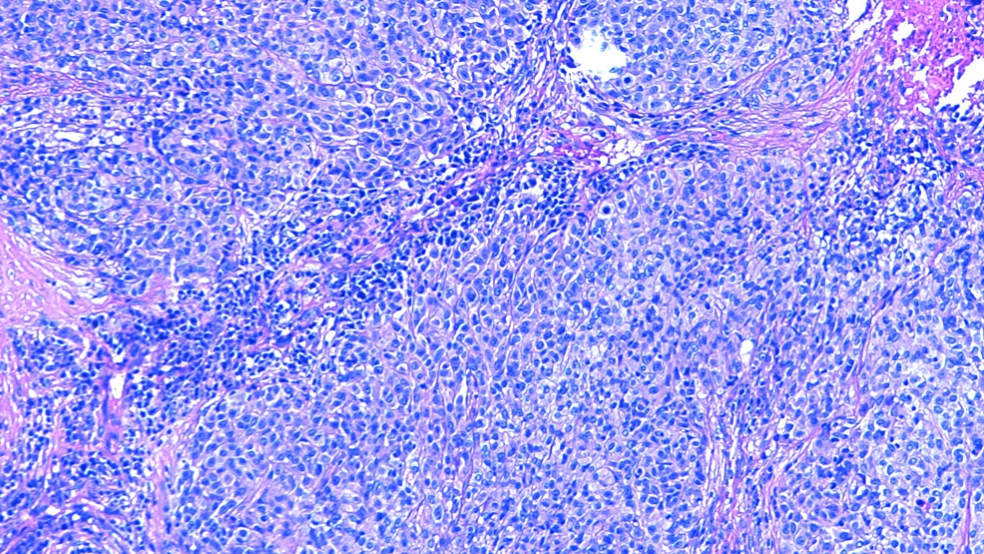
H&E staining that demonstrates widespread histological metastatic characteristics such as disorganized growth with invasion of the basement membrane, magnification 100x. H&E: Hematoxylin and eosin.

**Figure 6:**
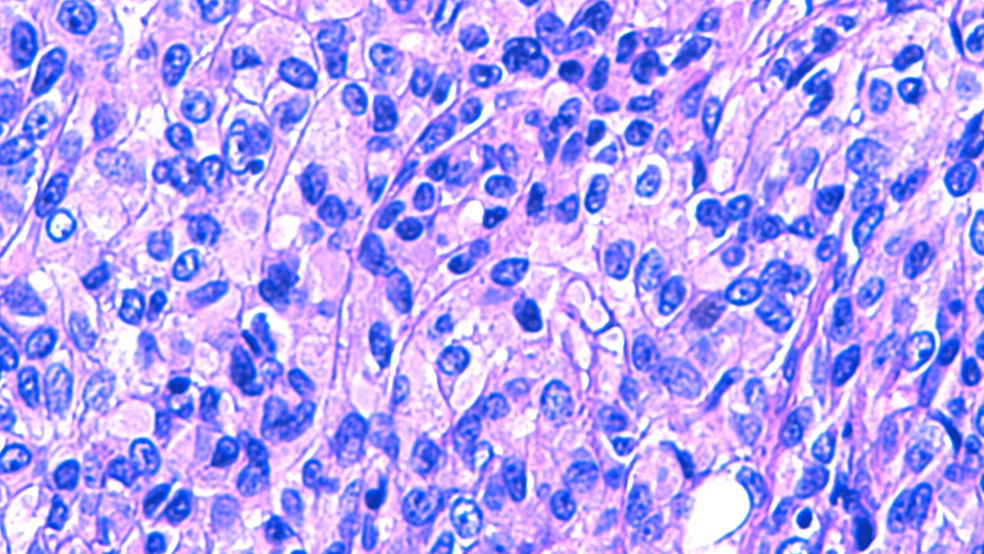
H&E staining demonstrating nuclear polymorphism and disorganized growth, magnification 500x. H&E: Hematoxylin and eosin.

**Figure 7:**
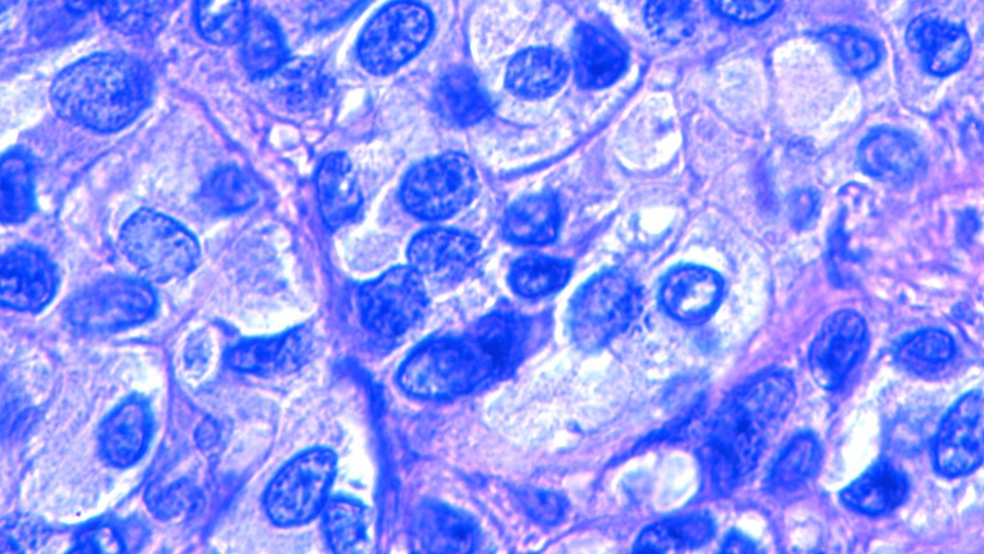
H&E staining highlighting the high nuclear to cytoplasmic ratio, magnification 1000x. H&E: Hematoxylin and eosin.

**Figure 8:**
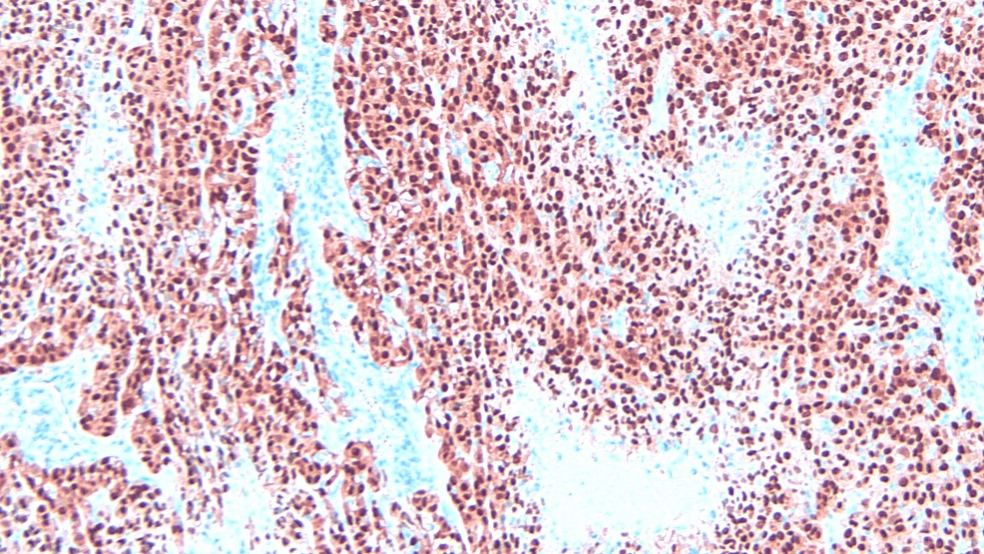
Sox staining with invasion of the basement membrane, magnification 100x.

**Figure 9:**
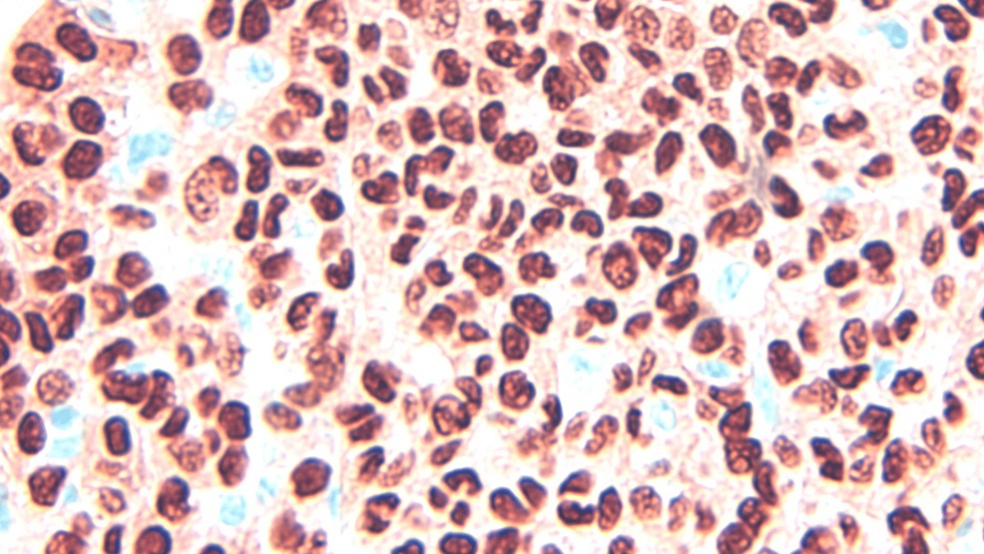
Sox staining with disordered growth and pleomorphism, magnification 500x.

**Figure 10:**
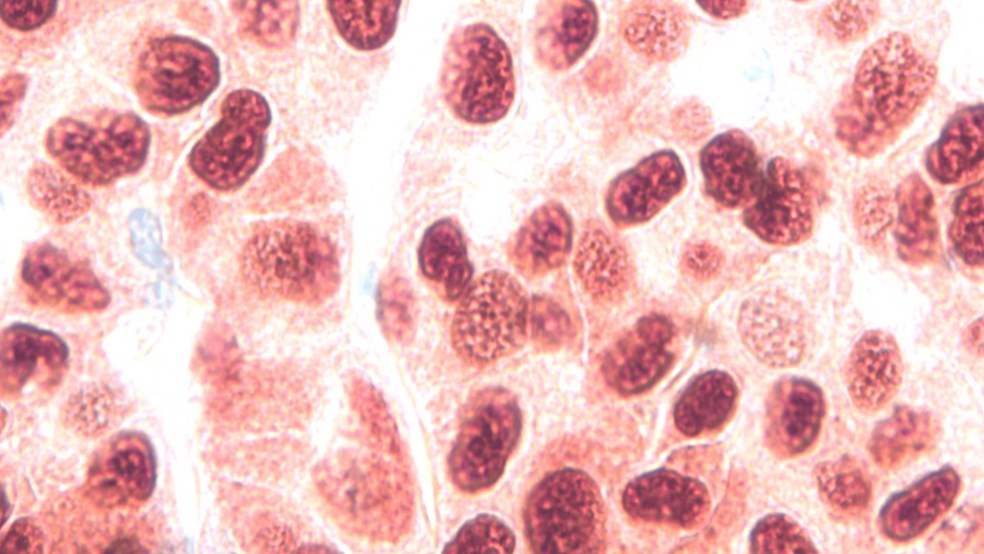
Sox staining demonstrating the high nuclear to cytoplasmic ratio and high mitotic activity, magnification 1000x.

Histology results revealed metastatic melanoma with hematoxylin and eosin (H&E) staining (Figures 5-7), stating the pathology reports were specific and in accordance with histological image findings. Flow cytometry revealed the tumor cells were exhibiting strong S100 and Sox10 positive immunostaining (Figures 8-10). The tumor cells were negative for GATA3, E-cadherin, estrogen and progesterone receptor, CD68, CD45, and broad-spectrum cytokeratin. These findings strongly support the diagnosis of metastatic melanoma of the left axilla. 

## Discussion

Melanoma metastasis to the breast is a rare phenomenon that represents approximately 1.3-2.7% of all malignant breast tumors and can mimic primary breast malignancies both clinically and radiologically [6,7]. The diagnostic challenge lies in differentiating between primary breast cancer and metastatic lesions. Clinically, many of these breast lesions present as a single lump in the upper outer quadrant of the breast. This region of the breast has a higher concentration of glandular tissue and blood circulation, making it a nidus for hematogenous spread of malignancy [8]. In such cases, serologic studies are important in differentiating the etiology. In this patient’s case, clinical history and immunohistochemistry played a pivotal role in confirming the diagnosis. 

Radiologically, malignant breast masses have distinct features based on their molecular subtype. Triple-negative breast tumors tend to be non-calcified with circumscribed margins, while luminal and HER2-positive breast cancer subtypes tend to be irregular with spiculated margins and pleomorphic calcifications, respectively [9]. Unfortunately, metastatic disease of the breast does not show one specific radiologic pattern and can simulate any of these primary breast tumors [7,8]. In our case, the patient presented with a hypoechoic mass with a circumscribed wall and irregular borders, showing mixed features that can be seen in triple-negative and HER2-positive breast tumors. 

The clinical presentation of the axillary mass, in this case, is intriguing, as the patient did not present with any other cutaneous lesions or systemic symptoms commonly associated with metastatic disease, such as unintentional weight loss or bone pain. This absence of other skin lesions or distant metastases highlights the potential for isolated regional spread, emphasizing the need for comprehensive evaluation even in the absence of typical signs of advanced disease. The unique nature of this case underscores the importance of maintaining a high suspicion for metastasis in patients with a history of malignant melanoma, even when the classic symptoms are absent.

The pathologic evaluation of nodal metastases is not certain as there is no clear marker for the diagnosis of melanoma. A combination of immunohistochemical stains must be used to achieve better sensitivity and specificity. The S100 marker that was previously stated serves as the most sensitive marker of melanocytic differentiation as it is found diffusely in all primary and secondary malignant lesions, however, it is not specific for melanoma cells as it stains other cell types [10,11]. Therefore, the incorporation of other markers must also be considered. The Sox10 marker is helpful for the diagnostics of melanoma as it serves as a nuclear transcription factor in the differentiation of neural crest progenitor cells to melanocytes [12].

Metastasis in the breast has been reported as being a poor prognostic indicator. The survival of a patient with breast metastasis from primary melanoma in four case series was 10 months [9]. Current breast cancer screening guidelines are yearly for women post-diagnostic mammogram, six months for women post-radiotherapy, and annual mammogram for women ages 40-75 with discontinuation of annual mammograms after 75 years of age [13]. Therefore, even though it is not explicitly stated in the mammogram guidelines, patients with a history of malignant melanoma that presents with any mass found, regardless of benign-appearing features on mammogram, should be further evaluated.

## Conclusions

We report a case of a 50-year-old woman with cutaneous malignant melanoma of the breast with metastatic spread, despite excision and closure of the primary malignancy. This hypoechoic left axillary mass was biopsied and found to have positive immunostaining congruent with cutaneous malignant melanoma. This case serves to add to the literature on the metastatic spread of cutaneous malignant melanoma of the breast and to help educate on how they can be distinguished from primary breast cancer. In addition, it highlights the importance of continuous patient-specific oncology surveillance (i.e., colonoscopy, mammogram, lung cancer screening, etc.) after the surgical treatment of cutaneous malignant melanoma due to potential metastatic spread and the significance of patient education.
